# Identification of an Identical *de Novo* SCAMP5 Missense Variant in Four Unrelated Patients With Seizures and Severe Neurodevelopmental Delay

**DOI:** 10.3389/fphar.2020.599191

**Published:** 2020-12-18

**Authors:** Xianru Jiao, Manuela Morleo, Vincenzo Nigro, Annalaura Torella, Stefano D’Arrigo, Claudia Ciaccio, Chiara Pantaleoni, Pan Gong, Katheryn Grand, Pedro A. Sanchez-Lara, Joel Krier, Elizabeth Fieg, Andrew Stergachis, Xiaodong Wang, Zhixian Yang

**Affiliations:** ^1^ Department of Pediatrics, Peking University First Hospital, Beijing, China; ^2^ Telethon Institute of Genetics and Medicine, Naples, Italy; ^3^ Department of Precision Medicine, University of Campania “Luigi Vanvitelli”, Naples, Italy; ^4^ Developmental Neurology Unit, Fondazione IRCCS Istituto Neurologico Carlo Besta, Milan, Italy; ^5^ Department of Pediatrics, Medical Genetics, Cedars-Sinai Medical Center, David Geffen School of Medicine, University of California, Los Angeles, Los Angeles, CA, United States; ^6^ Brigham and Women’s Hospital, Boston, MA, United States; ^7^ Cipher Gene Ltd., Beijing, China

**Keywords:** epilepsy, Secretory carrier membrane protein 5, developmental delay, autism spectrum disorder, congenital deformity

## Abstract

**Objective:** To establish and broaden the phenotypic spectrum of secretory carrier membrane protein (*SCAMP5)* associated with epilepsy and neurodevelopmental delay.

**Methods:** A Chinese patient was identified at the First Hospital of Peking University, and the three unrelated patients were recruited from two different countries (Italy and United States) through GeneMatcher. *SCAMP5* pathogenic variants were identified by whole exome sequencing; clinical data of the patients were retrospectively collected and analyzed.

**Result:** The onset age of seizures was ranged from 6 to 15 months. Patients had different types of seizures, including focal seizures, generalized tonic-clonic seizures and tonic seizure. One patient showed typical autism spectrum disorder (ASD) symptoms. Electroencephalogram (EEG) findings presented as focal or multifocal discharges, sometimes spreading to generalization. Brain magnetic resonance imaging (MRI) abnormalities were present in each patient. Severe intellectual disability and language and motor developmental disorders were found in our patients, with all patients having poor language development and were nonverbal at last follow-up. All but one of the patients could walk independently in childhood, but the ability to walk independently in one patient had deteriorated with age. All patients had abnormal neurological exam findings, mostly signs of extrapyramidal system involvement. Dysmorphic features were found in 2/4 patients, mainly in the face and trunk. All four unrelated patients were found to have the same heterozygous pathogenic *SCAMP5 de novo* variant (p. Gly180Trp).

**Conclusion:** Epilepsy, severe developmental delay, abnormal neurological exam findings, with or without ASD or variably dysmorphic features and were common in patients with *SCAMP5* variant. The onset time and type of seizure varied greatly. The EEG and brain MRI findings were not consistent, but diverse and nonspecific. The motor ability of patients with heterozygous *SCAMP5* variant might have a regressive course; language development was more severely affected.

## Introduction

Secretory carrier membrane proteins (SCAMPs) are widely distributed integral membrane molecules implicated in regulating vesicular transport (Law et al., 2012). *SCAMP* 1–4 are expressed ubiquitously, whereas *SCAMP5* is known to be brain specific and is present in high levels in synaptic vesicles (SVs), and the function of SCAMP5 for vesicle transport is selective (Fernández-Chacón and Südhof, 2000; Park et al., 2018). The human *SCAMP5* maps to chromosome 15q. Previously, there have been cases of patients with a chromosomal microdeletion of 15q leading to a series of neurological diseases (Sharp et al., 2007; Klopocki et al., 2008; Castermans et al., 2010; Ahram et al., 2017; Huynh et al., 2018). In 2010, *SCAMP5* was studied as a candidate gene for autism spectrum disorder (ASD) (Castermans et al., 2010). However, no disease-associated human *SCAMP5* point mutations were reported until 2020, when Hubert et al. reported two unrelated patients with an identical heterozygous *SCAMP5* variant (p. Gly180Trp) who had ASD, intellectual disability, and seizures (Hubert et al., 2020). Studies in a *Drosophila melanogaster* model have shown the pathogenicity of that variant (Hubert et al., 2020). Zhang et al. subsequently identified a homozygous variant of *SCAMP5* (p. Arg91Trp) in Chinese siblings with pediatric epilepsy and juvenile Parkinson’s disease (Zhang et al., 2020). *SCAMP5* variant knock-in mice showed typical early-onset epilepsy similar to that of humans (Zhang et al., 2020). The genetic pattern and specific phenotype of *SCAMP5* in human disease are unknown to date due to the limited numbers of reported cases.

Here, we present four unrelated patients with the identical *de novo SCAMP5* variant (p. Gly180Trp) from three countries, including detailed descriptions of initial clinical presentations and long-term follow-up (ranged from 1 to 33 years).

## Patients and Methods

### Ethics Statement

This study was approved by the respective local Ethic Committees (Patient 1: Biomedical Research Ethical Committee of Peking University First Hospital, approval number: 2016-1135; Patient 2: local Ethical Committee of Federico II University of Naples; Patient 3: Cedars-Sinai Medical Center; Patient 4: Brigham and Women’s Hospital). The individuals or their parents in this manuscript have been given the written informed consent to publish the case details.

### Patients

Patient 1 was recruited from the First Hospital of Peking University. Additionally, three individuals (patients 2, 3, 4) with the identical *de novo* variant in *SCAMP5* were connected to this study through GeneMatcher (Sobreira et al., 2015). The results of electroencephalogram (EEG), magnetic resonance imaging (MRI), neurological examination findings, biochemical studies, plasma amino acids and urine organic acids test in four patients were respectively collected in First Hospital of Peking University, Telethon Undiagnosed Diseases Program, Cedars-Sinai Medical Center, and Brigham and Women’s Hospital. Neurodevelopmental assessment was performed according to intelligence tests (Wechsler or Gesell intelligence scales) or clinical judgment and parents’ questionnaires.

### Genetic Analysis

DNA (3 µg) extracted from peripheral blood from probands and their parents was analyzed using whole-exome sequencing (WES) with standard protocol (Hubert et al., 2020). Sequence variants were checked with population databases gnomAD (http://gnomad.broadinstitute.org/) and evaluated using Polyphen2, SIFT, and Mutation Taster. The pathogenicity of variants were interpreted according to the American College of Medical Genetics (ACMG) guidelines (Richards et al., 2015). The variants were further confirmed by Sanger sequencing.

## Results

Clinical features of affected individuals with *SCAMP5* variants G180W were summarized in Tables 1, 2.

**TABLE 1:** Demographic and clinical manifestations of affected individuals with *SCAMP5* variant G180W

	Patient 1	Patient 2	Patient 3	Patient 4	Hubert et al. patient 1	Hubert et al. patient 2
Origin	Chinese	Italian	American	American	Caucasian	Maghrebian
Current age/Sex	2 y 3 m/M	8 y 4 m/M	2 y 6 m/F	32 y/F	10 y/M	8 y 6 m/M
Gestation	At term	At term	At term	10 days post-term	At term	37 weeks
Birth history	Unremarkable	Unremarkable	Unremarkable	Late decelerations during labor, a low forceps delivery	Unremarkable	Intrauterine growth retardation
Age at seizure onset	15 m	11 m	12 m	6 m	30 m	33 m
Seizure type	FS	TS, GTCS	GTCS	FS, GTCS	Absences and atonic seizures, MS, GTCS	n.a.
Evolution	Not treated	Under control at 12 months	Under control at 22 months	Under control at about 22 years	Under control at 7 years	Not treated
Other clinical feature	No	Motor stereotypes, rare aggressiveness outburst	No	Overweight (BMI = 29)	Attention deficit, hyperactivity, stereotypies, autistic features	Aggressiveness, hyperactivity, stereotyped swinging Movements
Psychomotor development	Severe delay	Severe delay	Severe delay	Severe delay	Severe delay	Severe delay
Walking	1 y 5 m	3 y 10 m	No	4 y	3 y	2 y 6 m
Speech	No	No	No	No	No	No

FS, focal seizure; TS, tonic seizure; GTCS, generalized tonic-clonic seizures; MS, myoclonic seizure; n.a., not available; m, month; y, year.

**TABLE 2:** EEG, brain MRI, neurological examinations, and dysmorphic features of affected individuals with *SCAMP5* variant G180W

	Patient 1	Patient 2	Patient 3	Patient 4	Hubert et al. patient 1	Hubert et al. patient 2
Current age/Sex	2 y 3 m/M	8 y 4 m/M	2 y 6 m/F	32 y/F	10 y/M	8 y 6 m/M
Interictal EEG	Frontal-temporal region discharges, sometimes spreading to generalization and mild diffuse background slowing	Multifocal bilateral discharges	Discharges in bilateral frontal-temporal and mild diffuse background slowing	n.a.	Frontal spikes and spike waves during sleep	Global slowing of background activity during drowsiness and sleep
Brain MRI	Age 1: Left choroid fissure cyst	Age 5: Temporal mesial bilateral hypotrophy, especially affecting the hippocampus, aspecific white matter anomalies in peritrigonal and periventricular regions, diffuse supratentorial sulci enlargement, posterior corpus callosum thinning, and bilateral signal alterations in globus pallidus, substantia nigra, and dentate nucleus.	Age 1: Periventricular cyst	Age 7: Thin corpus callosum, decreased white matter and mild dilation of lateral ventricles, subarachnoid spaces, and cerebellar fissures; age 13: Stable appearance of atrophic findings from age 7. New bilateral foci of FLAIR hyperintensity in periatrial white matter and focally in posterior limbs of internal capsules, likely new due to technical imaging differences; age 31: Slight atrophy of periventricular horns and thinning of corpus callosum.	Diffuse hyperintensity of the white matter, thin corpus callosum, mesial temporal sclerosis	Diffuse hyperintensity of the white matter, enlarged ventricules, mesial temporal sclerosis
Neurological exam	Dystonic postures while walking	Drooling, global hypotonia with limbs hyperreflexia, proximal limbs hyperkinesia, dystonic postures while walking	Hypotonia	Mild tremor, gait abnormalities	Ataxic gait, with trunk hypotonia, absent deep tendon reflexes, choreic delicate movements	Ataxic gait, global hypotonia
Facial dysmorphism	No	Progressive microcephaly, mildly narrow, depressed nasal bridge with broader tip, flat philtrum, large mouth with upper lip eversion, and protruding ears	No	Hypotelorism, bulbous nasal tip, downturned mouth, mild prognathic, mild anterior hairline elevation	n.a.	n.a.
Other dysmorphism	No	Scoliosis	No	Scoliosis	Varus equus	Varus equus

EEG, electroencephalogram; MRI, magnetic resonance imaging; n.a., not available; m, month; y, year.

### Seizures, Electroencephalogram and Brain Magnetic Resonance Imaging Information

In total, four patients from three different countries were enrolled, including two males and two females. Among the four patients derived from four unrelated families, three patients were born after a normal pregnancy and uneventful delivery. Patient 4 was born 10 days post-term. Delivery was complicated by late decelerations during labor, occipital posterior presentation and a low forceps delivery, though Apgar scores were 9. All patients had seizures and the age of onset was ranged from 6 to 15 months. Patients had different types of seizures, including generalized tonic-clonic seizures (GTCS, 3/4), focal seizures (2/4), and tonic seizure (1/4). As for patient 1, the first seizures occurred at the age of 1 year and 3 months. Four days later, the child experienced two further episodes in the same day. After that, no convulsion was observed up to the last follow-up (2 years and 3 months) and he was not treated with antiepileptic drugs (AEDs). The seizures of patient 2 and patient 3 were controlled by AEDs at the age of 12 and 22 months, respectively. As for patient 4, from the first episode to the age of 22, recurrent epileptic seizures were consistently experienced, although treated with AEDs. However, after a hysterectomy to treat menorrhagia (around the age of 22), she was seizure free for over 10 years, and her epilepsy continues to be managed with valproic acid. Up to the last follow-up, all patients showed seizure free. In addition to seizures, patient 2 sometimes showed motor stereotypes and rare aggressiveness outburst.

**FIGURE 1:**
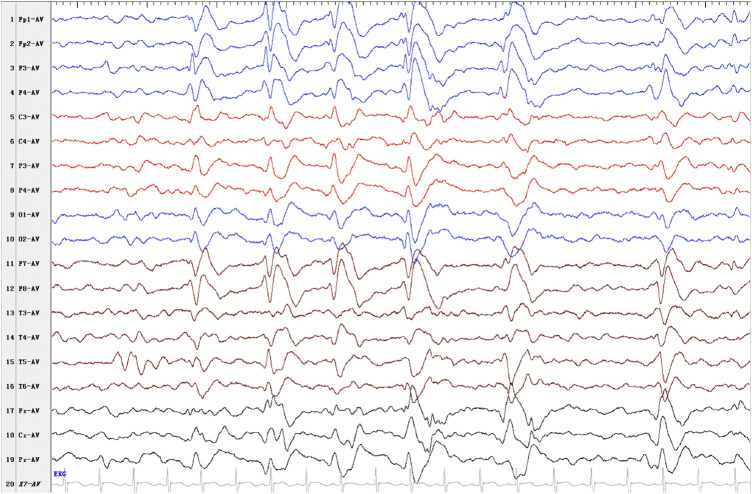
Electroencephalogram (EEG) outcome of patient 1. Interictal EEG presented as discharges in frontal-temporal and midline regions, sometimes spreading to generalization at the last follow-up (2 years and 3 months).

**FIGURE 2:**
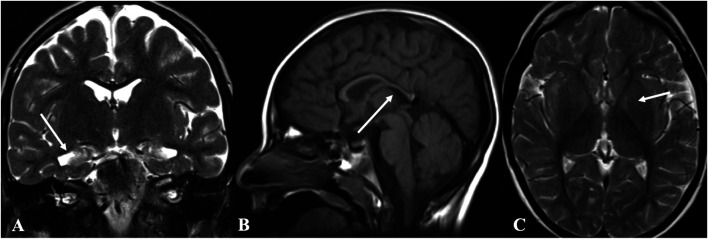
Brain magnetic resonance imaging (MRI) of patient 2. Brain MRI demonstrated temporal mesial bilateral hypotrophy (arrow in **A**, coronal T2w), especially affecting the hippocampus, aspecific white matter anomalies in peritrigonal and periventricular regions, diffuse supratentorial sulci enlargement, posterior corpus callosum thinning (arrow in **B**, sagittal T1w), and bilateral signal alterations in globus pallidus (arrow in **C**, axial T2w), substantia nigra, and dentate nucleus.

EEG findings were obtained from three patients. Patient 1: initial EEG examination showed frontal-temporal and midline regions discharges, sometimes spreading to generalization and mild diffuse background slowing. At the last follow-up (2 years and 3 months), the EEG had no significant change (Figure 1). Patient 2: several EEGs demonstrated the presence of multifocal bilateral epileptic anomalies. At the last follow-up (8 years and 4 months), EEG presented diffuse and discontinuous epileptic activity, and short series of focal frontal-central left anomalies. Patient 3: EEG at the age of 12 months showed bilateral frontal-temporal discharges and mild diffuse background slowing. At the last follow-up (2 years and 6 months), EEG showed only slowing in the background without discharges. Brain MRI abnormalities were present in each patient. Brain MRI of patients 1 and 3 at 1 year old showed cyst in left choroid fissure and periventricular, respectively. Brain MRI of patient 2 demonstrated temporal mesial bilateral hypotrophy, especially affecting the hippocampus, aspecific white matter anomalies in peritrigonal and periventricular regions, diffuse supratentorial sulci enlargement, posterior corpus callosum thinning, and bilateral signal alterations in globus pallidus, substantia nigra, and dentate nucleus (Figure 2, at the age of 5). Brain MRI of patient 4 at ages 7, 13, and 31 have demonstrated consistent findings including local atrophy and FLAIR white matter hyperintensities (Table 2).

### Neurodevelopment

All patients showed severe intellectual disability and developmental disorders, including motor and language development. Among them, three patients showed delayed motor development but could eventually walk independently at the last follow up, although with gait unsteadiness, and only one (patient 3) never reach autonomous walk. The motor development of patient 4 underwent a retrograde process. She crawled at 2 years and walked with a walker at 4 years, and required ankle foot orthosis braces from a very young age. She attained the ability to walk independently though at approximately age 30. She began to feel uncomfortable standing by herself and needed to hold onto something while standing or walking at all times and is now heavily reliant on a wheelchair (at the age of 32). At the last follow up, all patients were still backward in language development. They could understand simple instructions and speak a few words occasionally at the last follow up, which could not satisfy the normal communication.

### Neurological Examinations and Dysmorphic Features

Neurological exam of all patients revealed drooling, hypotonia, limbs hyperreflexia, proximal limbs hyperkinesia, dystonic postures, mild tremor and gait abnormalities.

Dysmorphic features were found in patient 2 and patient 4, mainly in the face and trunk. Facial deformities were varied, including progressive microcephaly, mildly narrow, depressed nasal bridge with broader tip, bulbous nasal tip, flat philtrum, large mouth with upper lip eversion, downturned mouth, mild prognathic, protruding ears, hypotelorism, and mild anterior hairline elevation. The other deformity was mainly scoliosis.

### Secretory Carrier Membrane Protein *5* Variant

All four patients underwent exome sequencing and the same *de novo SCAMP5* (c.538G>T p.Gly180Trp) heterozygous variant was identified. The variant was predicted to be “probably damaging,” “protein function affected” and “disease causing” to the protein structure by Polyphen-2, SIFT and Mutation Taster, respectively. The variant was absent in gnomAD population database, and classified as likely pathogenic according to ACMG guideline. The identical *SCAMP5* variant was reported in two patients by Hubert et al. (2020) with neurodevelopmental delay, ASD and epilepsy.

## Discussion

In 2020, Hubert et al. (2020) reported two unrelated patients with the identical heterozygous *de novo SCAMP5* variant (G180W) which was also identified in our cohort. Then, Zhang et al. (2020) identified a homozygous variant of *SCAMP5* (R91W) in Chinese siblings. To date, *SCAMP5* point mutations have been identified in four patients representing two different inheritance modes, and their pathogenicity has been confirmed by functional experiments (Hubert et al., 2020; Zhang et al., 2020). Using WES, we identified the identical *de novo* heterozygous *SCAMP5* variant (G180W) in four unrelated patients with a consistent phenotype, including epilepsy, severe developmental delay, abnormal neurological exams, and with or without ASD or variably dysmorphic features.

In 2010, a study identified *SCAMP5* as a candidate gene for ASD through detecting a *de novo* chromosomal translocation in ASD patients (Castermans et al., 2010). Both of the two patients reported by Hubert et al. exhibited characteristics of ASD, mainly in the form of stereotypical behaviors (Hubert et al., 2020). In our cohort, only patient 2 showed typical ASD symptoms, including motor stereotypes and rare aggressiveness outburst. Other patients have yet to find or obtain detailed information. In five cases with the novel 15q24 microdeletion syndrome that includes *SCAMP5*, ASD also had not been recorded (Sharp et al., 2007; Klopocki et al., 2008). Thus, ASD does not seem to be a universal feature of *SCAMP5* variants, even at the same mutation site.

Four patients here presented primarily with neurological abnormalities and began with epilepsy. The onset age of seizures ranged from 6 to 15 months, and three patients could be controlled with AEDs. In particular, patient 1 had only three episodes without AEDs therapy. Four cases reported internationally showed similar epileptic phenotypes (Hubert et al., 2020; Zhang et al., 2020). Seizures of two patients who had the same locus as ours occurred at 30 and 33 months, respectively (Hubert et al., 2020). Especially, in one patient, seizure was controlled at the age of seven, and the other did not require treatment (Hubert et al., 2020). The other two reported by Zhang et al. carrying homozygous variants both had seizures at 5 months, but the progression was unknown (Zhang et al., 2020). Between the reported and newly identified cases, there were various forms of seizures, including focal seizures, GTCS, tonic seizure, myoclonic seizures, absences and atonic seizures, and GTCS form was the most common. Thus, seizure was the common phenotype of patients with *SCAMP5* variants, but the onset time and forms varied greatly. Seizures of a small number of patients occurred only a few times and did not require medical intervention, and most of patients could be controlled by AEDs. In 2014, Zheng et al. reported increased susceptibility to heat-induced seizures in adult Drosophila with *SCAMP* deficiencies (Zheng et al., 2014). However, the susceptibility to heat-induced of epilepsy was not present in our cohort nor in previously reported patients (Hubert et al., 2020; Zhang et al., 2020). This might be because the genome of Drosophila contained only a single *SCAMP*, which differed from that of humans, so the phenotypes did not overlap completely. In our cohort, EEG findings presented as focal or multifocal discharges, sometimes spreading to generalization. Abnormalities of brain MRI were also present in each patient, and those were varied without specificity. Whereas, in previous literature, similar EEG and brain MRI patterns were thought to be present in patients with *SCAMP5* variants (Hubert et al., 2020). Thus, the EEG and MRI findings of patients with *SCAMP5* variants were not consistent, but diverse and nonspecific.

Severe intellectual disability and developmental disorders, including motor and language, were found in six patients, namely four patients here and two patients reported by Hubert et al. (2020) with same variant, whereas this was not seen in affected sibling with homozygous variants. Furthermore, studies in a *Drosophila melanogaster* model showed that the heterozygous variant resulted in developmental and locomotor defects that were absent in homozygous knock-in mutant mice (Hubert et al., 2020; Zhang et al., 2020). Therefore, different mutation site or inheritance patterns of *SCAMP5* may have different effects on neurodevelopment. For motor development, one of them in our cohort experienced a progressive gait deterioration. A similar disease course occurred in the patient reported by Hubert et al. (2020), whose condition gradually worsened and developed an ataxic gait with trunk hypotonia at the age of seven. By knocking out the *SCAMP* in Drosophila, an accelerated age-dependent decline in climbing ability were found in adults (Zheng et al., 2014). Therefore, the motor ability of patients with *SCAMP5* variants might be regressive with age. However, deterioration of motor ability was not observed in most patients. Thus, long-mid term follow-up of additional patients with *SCAMP5* variant was necessary to arrive at firm conclusions. All six patients with *SCAMP5* heterozygous variant had poor language development and were still unable to speak, either as reported in the literature or in our patients (Hubert et al., 2020). We can therefore conclude that language development is more severely affected than motor development.

Abnormal manifestations of neurological exam, especially in the extrapyramidal nervous system, are present in our cohort, especially in the two relatively older patients (ages 8 and 32), whereas the two younger patients only showed mild neuromotor abnormalities (at about the age of two). Acquired movement disorder features also have been described in some patients with *SCAMP5* heterozygous variant (at about the age of 8 and 10) (Hubert et al., 2020). Moreover, the two patients carrying *SCAMP5* homozygous variants also suffered from Parkinson’s disease at the age of 18 and 16, respectively (Zhang et al., 2020). The above symptoms suggest that the variants in *SCAMP5* might have an effect on neuromotor system function, especially in the extrapyramidal nervous system, and are age-related. Besides, some dysmorphic features could be seen in half of the patients in our cohort. Dysmorphic limbs features were also noted in two patients reported by Hubert et al. (2020) such as varus equus. Whereas, the two cases of homozygous *SCAMP5* mutation reported by Zhang et al. (2020) did not showed relevant abnormal descriptions. Thus, dysmorphic characteristics might be specific phenotypes to *SCAMP5* heterozygous variant, or even specifically to G180W.

In this study, all four patients carried the previously identified *de novo* heterozygous *SCAMP5* variants (G180W) (Hubert et al., 2020). Previously, both heterozygous and homozygous *SCAMP5* variants had been associated with human disease (Hubert et al., 2020; Zhang et al., 2020). In 2020, Zhang et al. (2020) reported homozygous R91W variants in siblings with early-onset epilepsy and Parkinson’s disease, while the parents with heterozygous R91W variants were normal. And that, the two patients did not show signs of ASD, intellectual disability, or other growth and developmental disorders (Zhang et al., 2020). It therefore appears that heterozygous and homozygous variants of *SCAMP5* are associated with distinct clinical phenotypes and inheritance patterns, and molecular characterization supports such a distinction. The two variants were located in different functional domains of the *SCAMP5*, with G180W located in the C-terminal tail and R91W located in the E peptide. Variants in different functional domains of *SCAMP5* might have different effects on gene functions. Therefore, further studies are needed to clarify the genetics and phenotype of *SCAMP5* in human.

It can be found from the description that *SCAMP5* is associated with a broad phenotype spectrum. *SCAMP5* is known to be brain specific and to be involved in vesicle transport (Fernández-Chacón and Südhof, 2000; Park et al., 2018). It may be due to its wide range of functions, leading to a wide range of clinical phenotypes. Studies have shown that the mutation of *SCAMP5* selectively activated excitatory synaptic signal transduction, leading to excitation-inhibition imbalance and recurrent seizures (Zhang et al., 2020). But the corresponding causes of other phenotypes need to be confirmed and linked with more research. Moreover, the phenotype spectrum and mutation sites also require more cases to be substantiated to determine whether the phenotype is associated with the mutation site or influenced by multiple factors.

## Conclusion

We identified the same *de novo* heterozygous *SCAMP5* variant in four unrelated patients with a consistent phenotype, including epilepsy, severe developmental delay, abnormal neurological exam findings, with or without ASD or dysmorphic features. The age of onset and type of seizures varied greatly. The EEG and brain MRI findings were not typical, but diverse and nonspecific. Developmental milestones of *SCAMP5* mutated patients show a more severe involvement of language skills, as language is generally absent, but patients may experience a regression of gait abilities. Dysmorphisms are present in some patients but not in all, even if carrying the same *SCAMP5* variant. Finally, variants of *SCAMP5* in different domains might result in different genetic and phenotypic patterns.

## Data Availability Statement

The original contributions presented in the study are included in the article/Supplementary Material, further inquiries can be directed to the corresponding authors.

## Ethics Statement

The studies involving human participants were reviewed and approved by The studies involving human participants were reviewed and approved by the Ethical Committee of Peking University First Hospital, local Ethical Committee of Federico II University of Naples, Cedars-Sinai Medical Center, and Brigham and Women’s Hospital. Written informed consent to participate in this study was provided by the legal guardians (parents) of the patient. Written informed consent was obtained from the legal guardians (parents) of the patient for the publication of any potentially identifiable images or data included in this article. Written informed consent to participate in this study was provided by the participants' legal guardian/next of kin.

## Author Contributions

XW and ZY conceptualized and designed the study, coordinated the study overall, and revised the manuscript; XJ co-designed the study, drafted the initial manuscript, and revised the manuscript; MM, VN, AT, SD’A, CC, CP, PG, KG, PAS-L, JK, EF, and AS helped to collect and summarize data and revised the manuscript. Telethon Undiagnosed Diseases Program contributed to the recruitment and whole exome sequencing (WES) processes of a patient described in the manuscript. All authors approve of the final revision of the article.

## Funding

This work was supported by National Nature Science Foundation of China (81771393), Beijing Municipal Science and Technology Commission (Z171100001017125), Beijing Natural Science Foundation (7202210), Capital’s Funds for Health Improvement and Research (2020-2-4077), Telethon Foundation (Telethon Undiagnosed Diseases Program, TUDP), Fondazione Pierfranco e Luisa Mariani (CM22).

## Conflict of Interest

Author XW was employed by the company Cipher Gene Ltd.

The remaining authors declare that the research was conducted in the absence of any commercial or financial relationships that could be construed as a potential conflict of interest.
