# Supplementary material for: Identification of an Identical de Novo SCAMP5 Missense Variant in Four Unrelated Patients With Seizures and Severe Neurodevelopmental Delay
Source: Front Pharmacol. 2020 Dec 18;11:599191. doi: 10.3389/fphar.2020.599191 (PMC7775611; doi:10.3389/fphar.2020.599191)
Supplement: Supplementary file 1 [file datasheet1.docx]

**Patient 1**

The male patient was the second child born to non-consanguineous Chinese parents. He has a healthy brother. The boy was born after a normal pregnancy and uneventful delivery. The first seizures occurred at the age of one year and three months, manifesting limbs stiffness, eyes slanting to the left or gaze fixed, purple lips and spittle, with spontaneous remission in two minutes. The child experienced two further episodes in the same day four days later. After that, no convulsion was observed up to the last follow-up (2 years and 3 months). He was not treated with antiepileptic drugs (AEDs). Brain MRI showed left choroid fissure cyst. Initial EEG examinations showed frontal-temporal and midline regions discharges, sometimes spreading to generalization and mild diffuse background slowing. At the last follow-up, the EEG had no significant change (Figure 1).

Within the first year of life, a global severely developmental delayed was also noted. He could walk unsteadily, understand simple instructions and speak a few words occasionally, which could not satisfy the normal communication. The boy was tested at the age of two years and three months with Gesell Development Diagnosis Scale and was found to be severely developmental delay (the last follow-up), and his adaptability was only equivalent to that of a child at 28.3 weeks. Dysmorphism feature was not noted.

**Patient 2**

The male patient is the only child of healthy non-consanguineous Italian parents. He was born at 40 weeks by spontaneous vaginal delivery, after an unremarkable pregnancy. Birth parameters were within the normal range, except for head circumference (32 cm, 9^th^ percentile). At the age of 11 months, generalized tonic seizures were observed, accompanied by oral cyanosis and perioral clonic movements. The initial seizures occurred during sleep and he subsequently experienced seizures also when awake. At the age of 12 months, the therapy of valproic acid was started with complete remission of the symptoms. Several EEGs demonstrated the presence of multifocal bilateral epileptic anomalies. At the last follow-up, EEG presented diffuse and discontinuous epileptic activity, and short series of focal frontal-central left anomalies. Brain MRI demonstrated temporal mesial bilateral hypotrophy, especially affecting the hippocampus, aspecific white matter anomalies in peritrigonal and periventricular regions, diffuse supratentorial sulci enlargement, posterior corpus callosum thinning, and bilateral signal alterations in globus pallidus, substantia nigra, and dentate nucleus (Figure 2).

Within the first year of life, a global severely developmental delayed was also noted, associated with progressive microcephaly, hypotonia and signs of extrapyramidal nervous system involvement. The boy acquired autonomous walking at the age of 3 years and 10 months, expressive language has never been accomplished. The kid could speak some vowels sounds and had limited non-verbal communication skills for basic needs. Sometimes he showed motor stereotypes and rare aggressiveness outburst. The boy was tested at the age of 3 years and 4 months with Griffith Mental Development Scales, scoring a globalization quotient (GQ) <24 months. Facial dysmorphisms were noted, including mildly narrow and depressed nasal bridge with broader tip, flat philtrum, large mouth with upper lip eversion, and protruding ears. Neurological exam revealed drooling, global hypotonia with limbs hyperreflexia, proximal limbs hyperkinesia and dystonic postures. He walked with flexed legs, valgus knees and sporadic lateral truncal deviation.

**Patient 3**

A 30-month-old female patient was the first child born to non-consanguineous American parents. She was conceived naturally and born at term gestation after a normal pregnancy and uneventful delivery. At the age of 12 months, she had her first generalized tonic-clonic seizure. Then, three episodes of generalized seizure occurred that lasted up to one minute each. EEG at this time was normal. The patient was started on levetiracetam therapy. Two days later, she had 3-4 episodes of generalized seizures. The combination treatment of levetiracetam, lorazepam, and fosphenytoin reduced seizure frequency but without a full control of the symptom. At the age of 14 months, the therapy of phenobarbital and levetiracetam was maintained, which controlled the seizures until 22 months of age when she had a few breakthrough seizures while trying to wear off phenobarbital. Medications were adjusted, and clobazam along with rufinamide were eventually added. She was also started on a ketogenic diet with the intent to wean her off of all medications. Up to now, she has been off of phenobarbital and rufinamide and remains seizure free for more than eight months while on the ketogenic diet. EEG at the age of 12 months showed bilateral frontal-temporal discharges and mild diffuse background slowing. At the last follow-up, EEG showed only slowing in the background without discharges. Brain MRI revealed a periventricular cyst at the age of 12 months.

At 30 months of age, she had severe motor and speech delays. She could crawl and walk with assistance. She was able to stand on her own for 5-10 seconds. In terms of language, she was babbling and could say “mama” without purpose. She previously said “dada”, but no longer was able to. Upon physical examination, does not show any dysmorphic facial features but has hypotonia, particularly affecting her lower extremities. There were initial concerns for hypothyroidism but repeat studies normalized without intervention.

A chromosome microarray identified two paternally inherited Variants of Uncertain Significance. A 44kb deletion (arr[GRCh37] 16p13.3(5078278_5123025)x1 pat and a 527 kb duplication arr[GRCh37] 16p13.11(15779481_16306653)x3 pat. Mitochondrial sequencing also identified a likely pathogenic MT-TY variant m.5835 G>A with very low heteroplasmy (approximated at 2 percent). Because of such low-level percentage, the clinical impact was difficult to interpret. Both parents are healthy and asymptomatic.

**Patient 4**

A 32-year-old female patient was the first child born to non-consanguineous American parents. She was born 10 days post-term. Delivery was complicated by late decelerations during labor, occipital posterior presentation and a low forceps delivery, though Apgar scores were 9. At six months of age, she developed focal seizures characterized by gaze deviation and choking episodes that later developed into generalized tonic-clonic seizures. She initially had several seizures per year but subsequently had improved control on phenobarbital. She was transitioned to carbamazepine but her white blood cell count dropped and so she was subsequently treated with valproic acid. Seizures became more frequent during puberty and she had 5-6 per year. However, after a hysterectomy to treat menorrhagia, she was seizure free for over 10 years. Her epilepsy continues to be managed with valproic acid. Brain MRI at ages 7, 13, and 31 have demonstrated consistent findings including local atrophy and FLAIR white matter hyperintensities.

She experienced developmental delay: she crawled at 2 years and walked with a walker at 4 years, and required AFO braces from a very young age. She attained the ability to walk independently though at approximately age 30. She began to feel uncomfortable standing by herself and needed to hold onto something while standing or walking at all times and is now heavily reliant on a wheelchair. She has not developed language other than isolated consonants and words such as “mama” as a child, no sign language. She attempts to interact readily with other people through grunts and reaching or grabbing. She went through menarche and adrenarche at an age consistent with her peers. She had severe scoliosis and required brace. Recently, she had onset of mild tremor. Facial dysmorphisms were noted, including hypotelorism, bulbous nasal tip, downturned mouth, mild prognathic, mild anterior hairline elevation.

Genetic evaluation as a young child included karyotype and Angelman/Prader-Willi loci deletion testing (FISH), and subsequently a chromosomal microarray in 2019 was negative.
