# Supplementary material for: Identification of an Identical de Novo SCAMP5 Missense Variant in Four Unrelated Patients With Seizures and Severe Neurodevelopmental Delay
Source: Front Pharmacol. 2020 Dec 18;11:599191. doi: 10.3389/fphar.2020.599191 (PMC7775611; doi:10.3389/fphar.2020.599191)
Supplement: Supplementary file 2 [file datasheet2.docx]

## Case information

| Patient ID | Age | Gender | Family history | Gene | Country | Mutation site/SV | Disease name | Phenotype | Symptom |
| --- | --- | --- | --- | --- | --- | --- | --- | --- | --- |
| 1 | 2y3m | M | no | SCAMP5 | China | Chr15:75018813; NM_001178111.2: c.538G>T p. (Gly180Trp) | - | Global developmental delay; epilepsy | Global developmental delay; epilepsy |
| 2 | 8y4m | M | no | SCAMP5 | - Italy |  | - | Global developmental delay; epilepsy;ASD;Dysmorphism | Global developmental delay; epilepsy;ASD;Dysmorphism |
| 3 | 2y6m | F | no | SCAMP5 | America |  | - | Global developmental delay;epilepsy | Global developmental delay;epilepsy |
| 4 | 32y | F | Don’t know | SCAMP5 | America |  | - | Global developmental delay;epilepsy; ;Dysmorphism | Global developmental delay;epilepsy; ;Dysmorphism |

Family history: Yes, No or don’t know;

家族病史：是否有家族病史；

Country: The patient's nationality or the patient's race;

国家：病人的国籍或人种；

Mutation site/SV: Nucleotide and protein change information or genotype;

突变/结构变异：核苷酸、蛋白质的改变或基因型信息/结构变异的位置；

Disease name: The exact disease the patient was diagnosed;

疾病名称：病人被确切诊断的疾病名称；

Phenotype: The phenotype of the disease;

表型：疾病的表型信息；

Symptom: The clinical symptom;

症状：临床症状信息
